# Knockdown of Brachyury Suppresses Breast Cancer Cell Proliferation and Migration via Targeting E2F3

**DOI:** 10.1155/2022/7913067

**Published:** 2022-11-22

**Authors:** Ming Chen, Jinyan Liu, Xiao Liang, Ying Huang, Zhengjie Yang, Pei Lu, Jun Shen, Keqin Shi, Huiheng Qu

**Affiliations:** ^1^Department of Orthopedic Surgery, The Affiliated Wuxi No. 2 People's Hospital of Nanjing Medical University, Wuxi, Jiangsu 214000, China; ^2^Department of Breast and Thyroid Surgery, The Affiliated Suzhou Hospital of Nanjing Medical University, Suzhou Municipal Hospital, Suzhou, Jiangsu 215002, China; ^3^Department of Anesthesiology, The Affiliated Wuxi No. 2 People's Hospital of Nanjing Medical University, Wuxi, Jiangsu 214000, China; ^4^Department of Ultrasonography, The Fifth People's Hospital of Suzhou, Suzhou, Jiangsu 215002, China; ^5^Department of Orthopeadic Surgery, The Affiliated Suzhou Hospital of Nanjing Medical University, Suzhou Municipal Hospital, Suzhou, Jiangsu 215002, China; ^6^Department of General Surgery, The Affiliated Wuxi No. 2 People's Hospital of Nanjing Medical University, Wuxi, Jiangsu 214000, China

## Abstract

Breast cancer is one of the most frequently diagnosed cancer in women and is the major cause of most cancer-related deaths. We previously reported that Brachyury, as a sensitive and specific marker, has been verified to involve in the process of carcinogenesis and progression of breast cancer, but the mechanism by which Brachyury promotes breast cancer cells proliferation and migration still remains less clear. In this study, we identified that Brachyury was markedly increased in breast cancer compared with the adjacent tissues. We have also shown that Brachyury knockdown could decrease the proliferation and migration capability in breast cancer cells both *in vitro* and *in vivo*. Finally, we found an important transcriptional factor, E2F3, which is a direct downstream target gene of Brachyury by chromatin immunoprecipitation (ChIP) analysis. Knockdown of E2F3 also decreased breast cancer cell proliferation and migration. Taken together, we reported that Brachyury may act as an oncogenic role in the progression of breast cancer by positively-regulating E2F3 expression.

## 1. Introduction

Breast cancer is the second most common and malignant cancer with greater than 266120 cases and approximately 40000 deaths each year in the United States [1, 2]. Breast cancer is a heterogeneous disease. According to the expression of progesterone receptor (PR), estrogen receptor (ER), ERBB2 receptor (HER2), and Ki-67, breast cancer can be divided into five groups: triple negative, HER2-enriched (non-luminal), Luminal B-like HER2+, Luminal A type, Luminal B type [3, 4]. Clinically, adjuvant therapy using endocrine therapy and surgical treatment has markedly reduced Luminal A primary breast tumors death. However, many patients will develop resistance to endocrine therapy [5]. In addition, a subset of triple-negative breast cancers features the deleterious expression of ER, PR, and HER2 and has a high risk of early recurrence and mortality compared with other molecular subtypes [6, 7]. Thus, new molecular markers and novel therapeutic targets need to be identified in order to better explain tumorigenesis and the progression of breast cancer.

The T-box gene family of transcription factors is known to participate in many aspects of embryogenesis in a wide variety of organisms, whose members share a highly conserved, sequence-specific DNA-binding domain of 180 to 200 amino acids known as the T-domain [8]. The first of the T-Box family molecularly characterized is Brachyury (official symbol: T-box transcription factor T (TBXT), Accession Number: HI638829.1) [8, 9], which is overexpressed in multiple solid tumors and plays a role in tumorigenesis and progression [8, 10, 11]. Interestingly, previous studies have reported that Brachyury is expressed in mesodermal precursor cells, gradually downregulated during late-stage embryos and is eventually undetectable in the majority of normal adulthood tissues [12]. More recently, Brachyury was described to be aberrantly overexpressed in several tumors, including lung [13–15], breast [11], prostate [16], colon [17], and testicular [18] cancer.

Notably, Brachyury has been reported as an independent biomarker of poor prognosis in these tumors. Many studies have verified that Brachyury was overexpressed in breast cancer tissues [19–21]. In addition, our previous studies reported that Brachyury plays a role in promoting cell proliferation in lung cells, which is consistent with our previous study of Brachyury on breast cancer cells [11]. Brachyury is expressed not only in primary tumors but also in metastatic lesions involving tumor-positive lymph nodes and distant metastases in breast cancer [11]. Furthermore, Brachyury has been associated with bone metastasis of breast tumors, stem cell TFs activation, and higher recurrence and metastatic spread, indicating that Brachyury may provide a new marker for aggressive breast tumors [11]. However, the impact and the underlying mechanisms of Brachyury on tumorigenesis of breast cancer, the primary breast tumor, remain to be determined.

In this manuscript, we report an investigation of the putative biological mechanism of Brachyury upon breast cancer tumorigenesis. We examined that Brachyury was excessively expressed in breast cancer tissues. We also investigated both the *in vitro* and *in vivo* effects of Brachyury on breast cancer proliferation and migration. We also performed chromatin immunoprecipitation (ChIP) to explore underlying downstream pathways regulated by Brachyury so as to provide an experimental and theoretical basis for further studies geared toward understanding the mechanism of breast cancer.

## 2. Materials and Methods

### 2.1. Patient Samples Collection

Human breast cancer and adjacent tissues were obtained from 25 patients who underwent surgery at the Suzhou Municipal Hospital of Nanjing Medical University. All participants provided written informed consent regarding this study, and ethical approval for the study was obtained from Nanjing Medical University. Participant information was fully protected.

### 2.2. Immunofluorescence

Immunofluorescent staining was performed to evaluate the expression of Brachyury in human breast cancer and adjacent tissues according to established procedures. Briefly, the paraffin-embedded human breast cancer and adjacent tissues were dewaxed and hydrated using a standard xylene-ethanol series. Antigen retrieval was carried out in 10 mM citrate buffer. After washing with Tris-buffer salt solution and 0.1% Triton X-100 (TBST), the sections were blocked with 1% BSA in TBST. Successively primary antibodies and corresponding secondary antibodies were added according to the protocol. Nuclei were counterstained with DAPI (Beyotime). The images were photographed using a confocal laser microscope (Zeiss LSM800, Carl Zeiss, Oberkochen, Germany).

### 2.3. Cell Culture and Transfection

MCF-7 and MDA-MB-231 (human breast cancer cell lines) were obtained from the Institute of Biochemistry and Cell Biology of the Chinese Academy of Sciences (Shanghai, China). All cells were maintained in Dulbecco's modified Eagle medium (DMEM, Thermo Fisher) supplemented with 10% fetal bovine serum (Gibco, New Zealand) and 1% penicillin/streptomycin (Invitrogen) and cultured at 37°C in a humidified incubator containing 5% CO_2_.

The interfering RNA (siRNA) sequences targeting Brachyury: (si-Brachyury#1)-5′-GCUGAACUCCUUGCAUAAG-3′; (si-Brachyury#2)-5′-GCUUAUCAGAACGAGGAGA-3′; the siRNA sequences targeting E2F3: (si-E2F3#1)-5′-GCGGUAUGAUACGUCUCUU-3′; (si-E2F3#2)-5′-GCAUCCACCUCAUUAAGAA-3′; the positive control siRNA: (si-GAPDH)-5′-UGACCUCAACUACAUGGUU-3′ and the negative control siRNA (si-NC)-5′-UUCUCCGAACGUGUCACGU-3′ were purchased from GenePharma (Shanghai, China). Brachyury, E2F3, and the negative control siRNAs were transfected into MCF-7 and MDA-MB-231 cells with Lipofectamine 2000 (Invitrogen, USA) according to the manufacturer's instructions. Sh-Brachyury and empty vector (GenePharma) were stably transfected into MDA-MB-231 cells. Cells were harvested for analysis at 48 h post-transfection.

### 2.4. Western Blot Analysis

Western blot analyses were performed with 20 *μ*g of protein from cell lysates. The cell lysates were resolved by 10% SDS-polyacrylamide gel electrophoresis (SDS-PAGE) and then transferred onto polyvinylidene difluoride membranes. After blocking, the membranes were incubated with the indicated primary antibodies (antiBrachyury and anti-*β*-Actin) overnight at 4°C. The next day, the membranes were washed and then incubated with horseradish peroxidase (HRP)-conjugated secondary antibodies for 1 hour at room temperature (RT). After washing, band signals were visualized using an ECL prime western blotting detection system. AntiBrachyury and Anti-*β*-actin purchased from cell signaling technology, Inc (CST). Anti-*β*-actin was used in parallel as the loading control, and experiments were repeated three times.

### 2.5. Cell Viability Assay

Cell viability was determined using a cell counting kit-8 kit (CCK8; Beyotime Biotechnology) at five time points (0, 24, 48, 72, and 96 h, respectively) in accordance with the manufacturer's protocol. After transfection with siRNAs, the MCF-7 and MDA-MB-231 cells were seeded at 2500 cells per well in 96-well plates. The optical density (OD) was measured at 450 nm wavelength with a microplate reader (Bio-Rad Model 680, Richmond, CA, USA). The experiment was performed in triplicate.

In the colony formation assay, MCF-7 and MDA-MB-231 cells were seeded into 6-well plates (800 cells/well) after transfection and maintained in proper media containing 10% FBS for two weeks. Then, the cell colonies were washed with PBS, fixed with methanol, and stained with 0.1% crystal violet (Sigma-Aldrich). Photographs were subsequently taken, and the process was performed in triplicate.

### 2.6. Cell Migration Assay

Cell migration capacity was evaluated via 24-well chambers with 8 *μ*m pore size. After 24 h of transfection, MCF-7 and MDA-MB-23 cells (4.5 × 10^4^ cells) were suspended in 300 *µ*l medium without serum and cultured in the upper transwell chamber. Meanwhile, the lower chamber was filled with 700 *µ*l medium supplemented with 10% FBS. Following 24 h of cultivation, the cells on the upper membrane were removed with a sterile cotton swab, whereas cells on the lower membrane surface were fixed with 4% paraformaldehyde and stained with 0.1% crystal violet. The number of cells was calculated under a microscope in five random fields. The experiment was conducted three times.

### 2.7. Xenograft Mouse Model

In brief, for in vivo growth assays, 1 × 10^6^ MDA-MB-231 cells transfected with sh-Brachyury or control were injected subcutaneously into the flanks of BALB/C-nu/nu athymic nude mice. The tumor size was measured using calipers every 3 d up to 15 d. After 15 d, the mice were sacrificed, and tumor volumes and weights were examined. The expression of Ki67 was examined using immunofluorescent staining. The animal study was approved by the Ethics Committee of Nanjing Medical University.

### 2.8. Chromatin Immunoprecipitation and Sequencing

The chromatin immunoprecipitation and sequencing (ChIP) assay was performed using a ChIP assay kit (Millipore, Billerica, MA, United States) following the instructions provided by the manufacturer. In brief, cells were harvested, washed, and cross-linked using 1% formaldehyde. Then, the DNA-protein complexes were isolated and sheared into fragments of 300–500 bp in length. The isolated chromatins were immunoprecipitated using an antiBrachyury antibody and normal rabbit IgG (negative control). Immunoprecipitated DNA was analyzed by real-time PCR using the following primers: the human E2F3 forward 5- ATTGTCAGCAGCAGCTTCCT-3, and reverse 5- GGGCCAAAAATAATCGGGGC-3; and the human GAPDH, forward 5′-AACCCAAACTAACAGTTGTCCCAA-3′and reverse 5′-ACTCCTTGGAGGCCATGTAGG-3′. The experiment was replicated three times.

### 2.9. Dual-Luciferase Reporter Assay

MCF-7 and MDA-MB-231 cells cultured in 96-well plates were transiently cotransfected with 50 ng of pGL3-E2F3 promoter or control vector and 30 ng of pcDNA3.0-Brachyury vector (GenePharma) for 24 h. After transfection, luciferase activity was detected using the Dual-Luciferase Reporter Gene Assay System (Promega, Madison, WI). The results were normalized based on the Renilla luciferase luminescence intensity (Promega-GloMax).

### 2.10. RNA Extraction and Quantitative Real-Time Reverse Transcription-PCR

Total RNAs were extracted from tissues as well as MCF-7 and MDA-MB-231 cells using RNA isolater Total RNA Extraction Reagent (Vazyme, Nanjing, Jiangsu, China). cDNA was synthesized by reverse transcription using a HiScript III RT SuperMix for qPCR (+gDNA wiper) kit (R323-01, Vazyme, Nanjing, China) following manufacturer-provided protocol. qRT-PCR was performed to quantify gene expression using the Applied Biosystems 7500 Real-Time PCR System and AceQ qPCR SYBR Green Master Mix (Q131, Vazyme, Nanjing, China). Results were normalized to 18sRNA expression and calculated based on the 2^–ΔΔ^CT method. The following primers were used: the human E2F3 forward 5′-ATTGTCAGCAGCAGCTTCCT-3′, and reverse 5′-GGGCCAAAAATAATCGGGGC-3′; and the human Brachyury forward 5′-GCTGGACCAATTGTCATGGG-3′; and reverse 5′-GGGTACTCCCAATCCTATTCTGAC-3′; and the human 18sRNA, forward 5′-AAACGGCTACCACATCCAAG-3′ and reverse 5′-CCTCCAATGGATCCTCGTTA-3′.

### 2.11. Statistical Analysis

GraphPad Prism software was used for statistical analysis. Data obtained by the Student's *t*-test or one-way ANOVA were presented as means ± standard deviation (SD) from at least three independent experiments. *p* values less than 0.05 were recognized as statistically significant.

## 3. Results

### 3.1. Brachyury Expression is Significantly Upregulated in Human Breast Cancer Tissues

**Figure 1:**
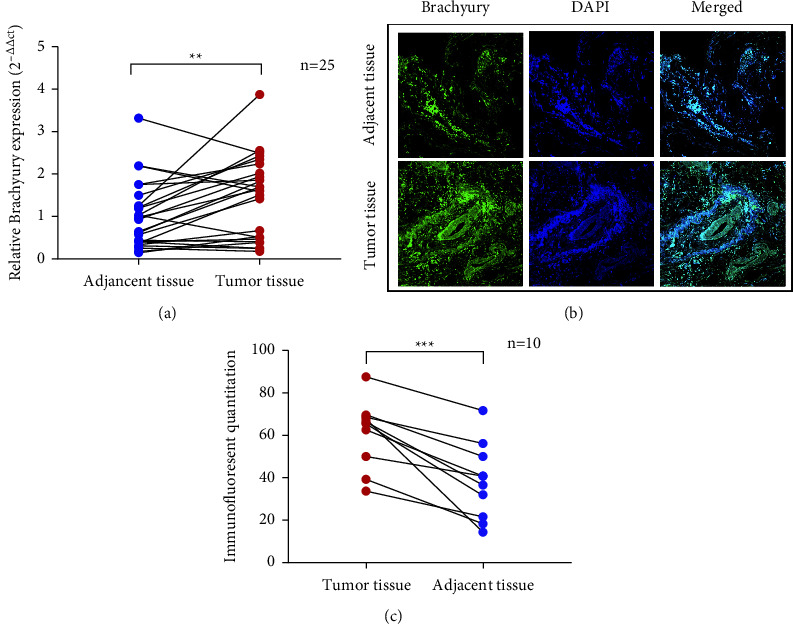
Relative Brachyury expression in breast cancer tissues. (a) The differential mRNA expression of Brachyury in breast cancer tumor tissues and adjacent tissues was detected by qRT-PCR (*n* = 25). Brachyury protein expression in human breast cancer tissues (b) detected by immunofluorescence (*n* = 10). Immunofluorescence staining for DAPI was used to stain nuclei. Original magnification 40×. Scales bars: 50 *μ*m. Analysis that represents the quantification of the intensity of immunofluorescence of Brachyury in human breast cancer (c). ^*∗*^*p* < 0.05, ^*∗∗*^*p* < 0.01, ^*∗∗∗*^*p* < 0.001, compared with adjacent tissue for each condition via Student's *t*-test.

To investigate the function of Brachyury in breast cancer, we first verified the different mRNA expression levels of it in breast cancer and adjacent tissues by RT-qPCR. As a result, the expression level of Brachyury mRNA in tumor tissues was higher than that in adjacent tissues, and there was a statistically significant difference (Figure 1(a)). After then, we compared the expression of Brachyury protein in breast cancer tissues by immunofluorescence. As shown in Figures 1(b) and 1(c), the average levels of Brachyury were higher in breast cancer tissues as compared to adjacent nontumor tissues.

### 3.2. Brachyury Knockdown Decreased Breast Cancer Cell Proliferation and Migration *in Vitro*

**Figure 2:**
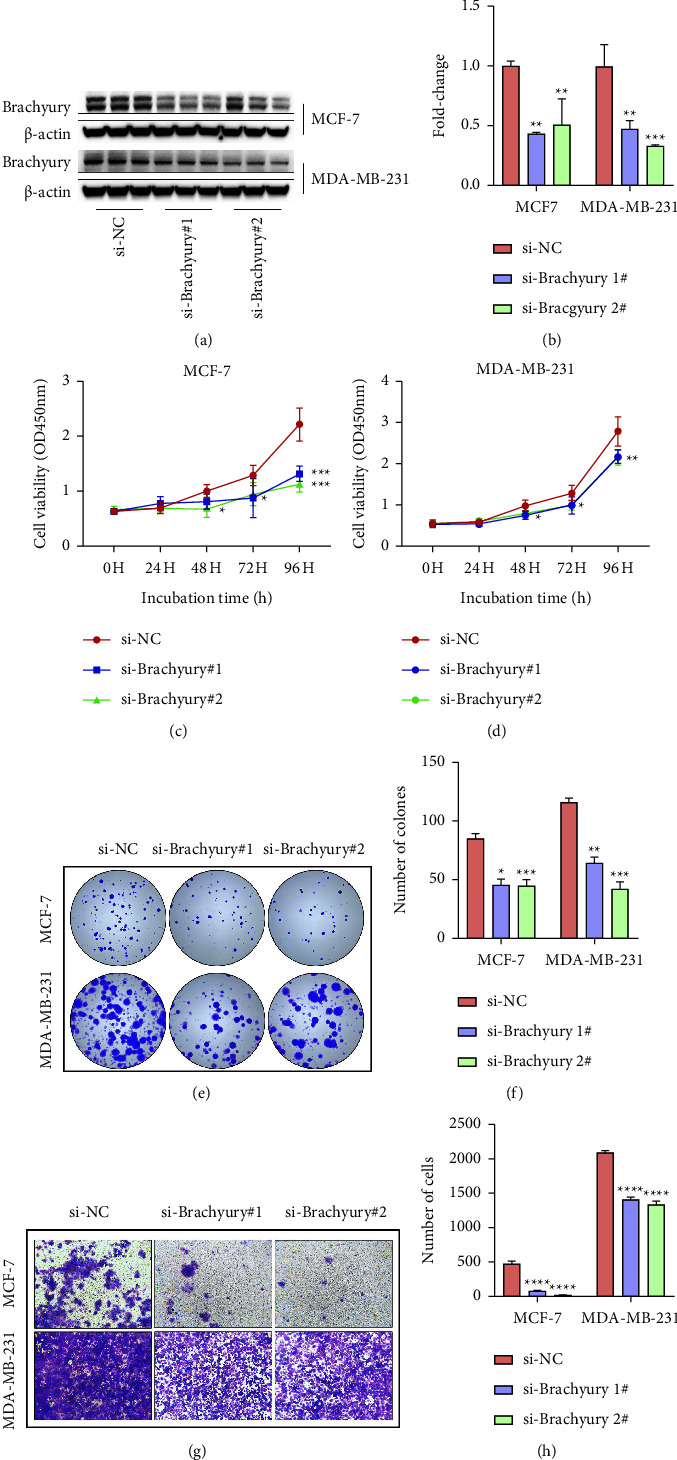
The effect of brachyury on breast cancer cells proliferation and migration in vitro. (a) Western blot analysis to confirm the siRNAs mediated knockdown of Brachyury in MCF-7 and MDA-MB-231 cells. (b) Quantification of (a). (c, d) CCK8 assays revealed that knockdown of Brachyury attenuated the growth of both MCF-7 and MDA-MB-231 cells up to 96 h compared with si-NC group. (e) Colony formation assays were performed to determine the proliferation ability of si-Brachyury-transfected MCF-7 and MDA-MB-231 cells. (f) Quantification of (e). (g) Transwell assays were performed to investigate the changes in migratory abilities of si-Brachyury-transfected MCF-7 and MDA-MB-231 cells. (h) Quantification of (g). ^*∗*^*p* < 0.05, ^*∗∗*^*p* < 0.01, ^*∗∗∗*^*p* < 0.001, ^*∗∗∗∗*^*p* < 0.0001.

To identify the role of Brachyury on the proliferation and migration capability of breast cancer cells *in vitro*, the triple-negative breast cancer cell line MDA-MB-231 and ER^+^/PR^+^/HER-2-luminal human breast cancer cell line MCF-7 as models for the triple-negative and the luminal A subtype of breast cancer were utilized for further study. Next, two independent siRNAs 1# and 2# were transfected into MCF-7 and MDA-MB-231 cells to knock down Brachyury expression levels. It was satisfactory that Brachyury was more efficiently diminished by siRNAs 1# and 2# (Figures 2(a) and 2(b)), which were selected for the next functional experiments. Obviously, CCK8 (Figures 2(c) and 2(d)) and colony formation assays (Figures 2(e) and 2(f)) *in vitro* showed that the proliferative ability of MCF-7 and MDA-MB-231 cells were decreased after Brachyury downregulation. Next, we examined the influence of Brachyury on the migration of breast cancer cells by transwell assay. Interestingly, the number of migrational cells was strongly reduced after Brachyury depletion in MCF-7 and MDA-MB-231 cells (Figures 2(g) and 2(h)). In summary, Brachyury knockdown could decrease the proliferation and migration capability in MCF-7 and MDA-MB-231 cells.

### 3.3. Silencing Brachyury Inhibits Breast Cancer Cells Proliferation *in Vivo*

**Figure 3:**
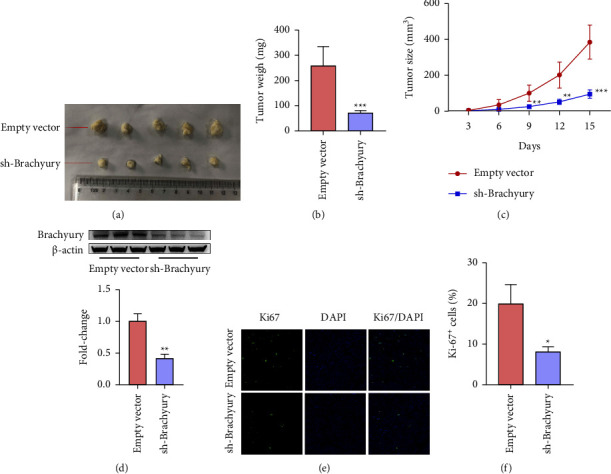
Silencing Brachyury inhibits MDA-MB-231cells proliferation *in vivo*. Brachyury knockdown or control MDA-MB-231 cells were subcutaneously injected into the nude mice. (a) Tumors were collected from the nude mice and photographed. (b) Tumor weights in the control group and Brachyury knockdown were calculated. (c) Tumor volumes were calculated after injection every 3 days. (d) The relative Brachyury expression level in the tumors derived from the control group and Brachyury-knockdown group by WB. (e, f) The expression of Ki-67 in the control group and Brachyury-knockdown group was performed by immunofluorescent assay. Scale bar = 50 *μ*m. ^*∗*^*p* < 0.05, ^*∗∗*^*p* < 0.01, ^*∗∗∗*^*p* < 0.001.

To further determine whether Brachyury could promote breast cancer cell proliferation *in vivo*, MDA-MB-231 cells stably transfected with sh-Brachyury or control empty vector were inoculated into nude mice. Tumor volumes were calculated after injection every three days. As expected, tumors in the Brachyury-depleted group showed a remarkable shrinkage, with significantly decreased tumor volumes and tumor weights compared with the control group (Figures 3(a)–3(c)). And, it is obvious that tumors derived from the sh-Brachyury group express lower levels of Brachyury (Figure 3(d)). Next, to investigate Brachyury's role in promoting cell proliferation *in vivo*, we performed immunofluorescent staining to measure the protein levels of Ki-67. The results presented that a marked decrease in the numbers of Ki-67-positive (Ki-67^+^) cells was observed in Brachyury-knockdown tumor tissues (Figures 3(e) and 3(f)), indicating Brachyury knockdown suppressed cell proliferation *in vivo*.

### 3.4. Brachyury Positively Regulates the Transcription of E2F3

**Figure 4:**
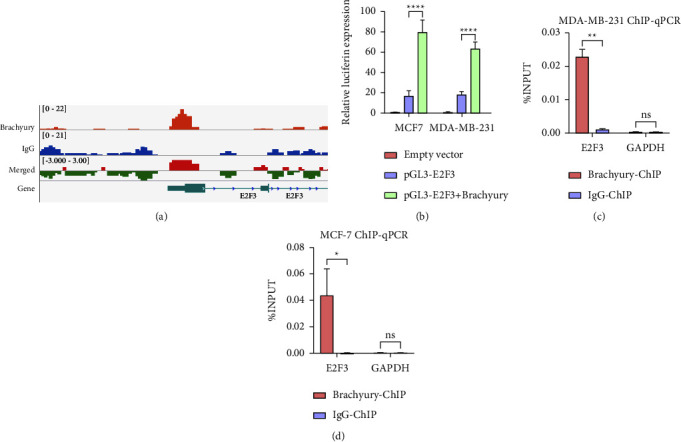
Brachyury positively regulates the transcription of E2F3. (a) Representative ChIP-seq peaks surrounding Brachyury target genes (E2F3). (b) Luciferase activity assays in MCF-7 and MDA-MB-231 cells transfected with pGL3-E2F3 and pcDNA3.1-BRY plasmids for 48 h DMSO combined with empty vector (EV) were used as the control. (c, d) ChIP-qPCR analysis of Brachyury-associated DNA sequences from the putative Brachyury-binding region of the E2F3 promoter in MCF-7 and MDA-MB-231 cells. The GAPDH gene was used as a negative control. ^*∗*^*p* < 0.05, ^*∗∗*^*p* < 0.01, ^*∗∗∗*^*p* < 0.001, ^*∗∗∗∗*^*p* < 0.0001.

Our previous studies have shown that Brachyury directly regulates the expression of SOX5 by binding to two motifs in its promoter region using ChIP analysis and reporter assays in MDA-MB-231 cells [11]. Through previous ChIP-seq data [11], we found that a representative example of the ChIP-seq peaks for E2F3 (E2F Transcription Factor 3, Accession Number: AF547386.1) is shown (Figure 4(a)). Next, we further detected the regulatory activity of Brachyury on the E2F3 promoter using dual-luciferase reporter assay and ChIP-PCR and found that the E2F3 promoter activity was significantly increased in MCF-7 and MDA-MB-231 cells cotransfected with the pGL3-E2F3 luciferase reporter vector and pcDNA3.1-Brachyury (Figure 4(b)). As expected, the Brachyury-binding peak sequence was precipitated with E2F3 in MCF-7 and MDA-MB-231 cells using ChIP-qPCR assay (Figures 4(c) and 4(d)). The findings indicated that Brachyury regulates the transcription of E2F3.

### 3.5. Knockdown of E2F3 Represses Cell Proliferation and Colony Formation of Breast Cancer Cells

**Figure 5:**
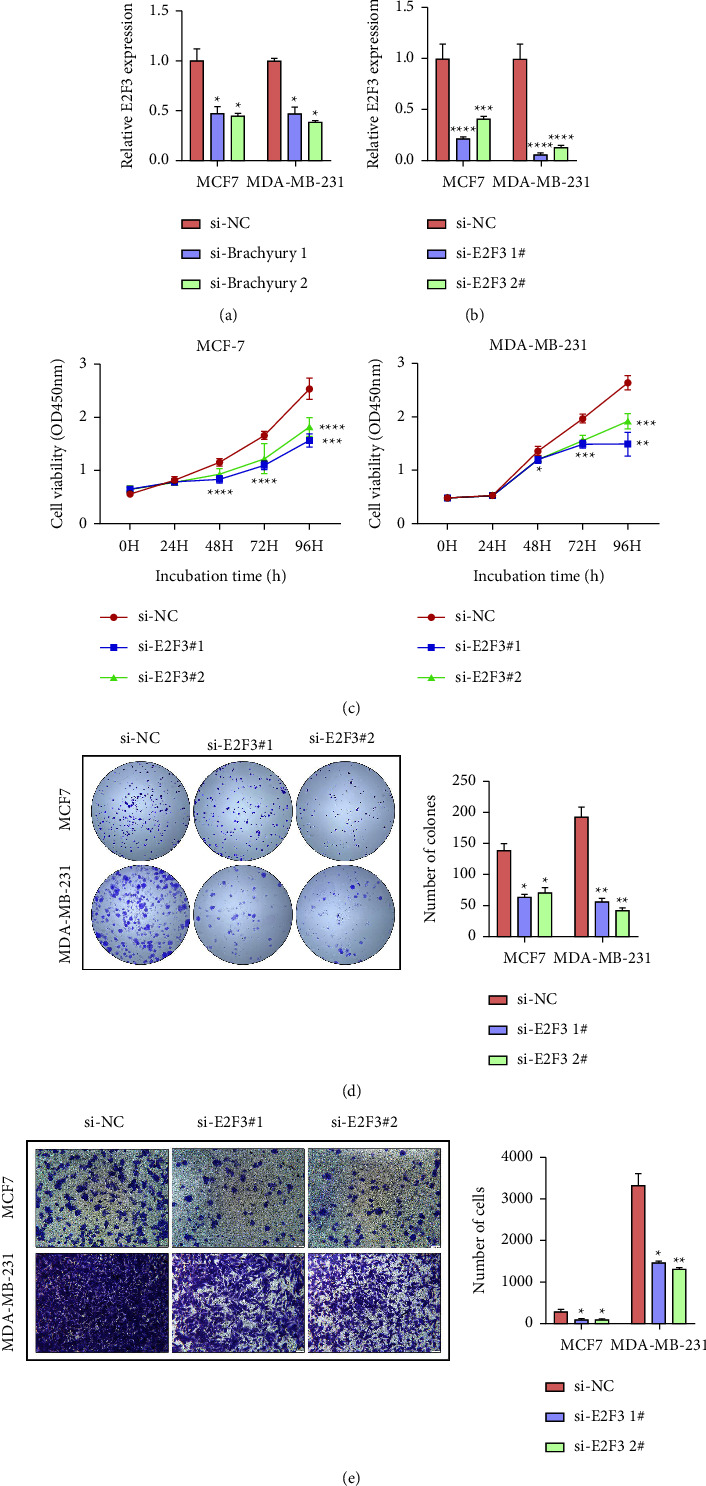
Effects of E2F3 on cell proliferation and migration in MCF-7 and MDA-MB-231 cells. The mRNA levels of E2F3 were detected after Brachyury (a) or E2F3 (b) downregulation in MCF-7 and MDA-MB-231cells. (c) CCK8 assays revealed that the knockdown of E2F3 attenuated the growth of both MCF-7 and MDA-MB-231 cells up to 96 h, compared with the si-NC group. (d) Colony formation assays were performed to determine the proliferation ability of si-E2F3-transfected MCF-7 and MDA-MB-231 cells. (e) Transwell assays were performed to investigate the changes in migratory abilities of si-E2F3-transfected MCF-7 and MDA-MB-231 cells. ^*∗*^*p* < 0.05, ^*∗∗*^*p* < 0.01, ^*∗∗∗*^*p* < 0.001, ^*∗∗∗∗*^*p* < 0.0001.

To confirm the regulation of Brachyury on E2F3 expression, si-Brachyury or si-E2F3 siRNAs were transfected into MCF-7 and MDA-MB-231 cells. As shown in Figure 5(a), E2F3 expression levels decreased after the knockdown of Brachyury. Considering the transcriptional regulation of E2F3 by Brachyury, we transfected with E2F3 siRNAs into MCF-7 and MDA-MB-231 cells, and the expression of E2F3 was indeed reduced (Figure 5(b)). Apparently, the knockdown of E2F3 decreased breast cancer cell proliferation (Figures 5(c) and 5(d)). Transwell assay revealed that after downregulating the expression of E2F3, the number of migrated MCF-7 and MDA-MB-231 cells was remarkably suppressed (Figure 5(e)). Taken together, these results demonstrated that Brachyury knockdown inhibited breast cancer cell proliferation and migration via interaction with E2F3.

## 4. Discussion

Breast cancer is the most common malignancy among women and is the major cause of most cancer-related deaths around the world [22]. Currently, the main unmet need for breast cancer is a better understanding of susceptibility genes and what drives invasiveness and metastasis, factors responsible for the lethality of this disease. Recently, Hu et al. [23] included 28 genes and 64,000 women from the United States to provide estimates of the prevalence and risk of breast cancer associated with pathogenic variants in known breast cancer-predisposition genes. They thought that these estimates can inform breast cancer testing and screening and improve clinical management strategies for women in the general population with inherited pathogenic variants in these genes [24]. The T-box protein Brachyury is a transcription factor that is widely expressed in multiple solid tumors and is associated with tumor aggressiveness and poor patient prognosis [25]. Our previous study has demonstrated that Brachyury and downstream target genes together involve in lung cancer cell cycle regulation by inducing accelerated transition through G2/M, promoting tumor cell proliferation and inhibiting apoptosis [15]. In addition, Brachyury has been proven to play an important role in promoting breast cancer cell progression [8, 11]. It has been reported that Brachyury is a susceptibility gene for breast cancer, which is positively correlated with the invasive and metastatic ability of breast cancer in vitro and with the risk of recurrence and distal metastasis in breast patients [11, 21]. However, the specific downstream target genes of Brachyury responsible for mediating its migration effects in breast cancer are still unclear. In the present study, we provide direct evidence that Brachyury is a direct target of E2F3 and is important for E2F3 to promote tumor proliferation and migration.

In this study, we found that the expression of Brachyury mRNA and protein was markedly increased in human breast cancer compared with the adjacent tissues (Figure 1). Brachyury knockdown could decrease the proliferation and migration capability in MCF-7 and MDA-MB-231 cells. Consistent with previous results, Brachyury is a significantly higher expression of mRNA and protein levels in breast cancer tissues compared with adjacent nontumor tissues [8, 11, 21]. What is more, Brachyury is involved in the invasion, migration, adhesion, and colonization of breast cancer [8, 26, 27], indicating that Brachyury plays an important role in the development of breast cancer. Brachyury is required for mesoderm formation and notochord development and has become an attractive target in the study of tumorigenesis and therapy [28]. Although various studies have investigated its role in breast cancer tumorigenesis and progression, a characterization of Brachyury's biological role in breast tumorigenesis is missing. Brachyury exerts its regulatory role by controlling the transcription of a large number of target genes [29]. Hotta et al. [30] showed that Brachyury's target genes encoded components for the regulation of the cell cycle and the production of ECM, multiple growth factors, and cytokines. Therefore, mechanistic dissection of Brachyury downstream target genes responsible for mediating its invasive effects will provide the rationale for utilizing Brachyury targeted immunotherapy for breast cancer.

Furthermore, E2F3 was identified as the transcriptional target of Brachyury using ChIP assays. This study provides direct evidence to support the inhibition of breast cancer cell proliferation and migration after knocking down Brachyury, mainly by targeting the E2F3 gene. E2F transcription factor 3 (E2F3) locates on chromosome 6p22, which is a family of transcription factors that regulate both cellular proliferation and the cell cycle process [31–33]. It is reported that the functions and transcriptional activity of E2F3 are altered in a variety of human malignancies, including lung, ovarian, bladder, gastric, and prostate cancers [34]. Given that both Brachyury and E2F3 could affect breast cancer progression, the relationship between Brachyury and E2F3 in breast cancer remains unclear. In this study, we found that Brachyury knockdown inhibited breast cancer cells proliferation and migration via interacting with E2F3 both in MCF-7 and MDA-MB-231 cell lines, suggesting that Brachyury exerts the biological function by regulating E2F3 genes in breast cancer.

Collectively, the present study indicated that Brachyury is a key regulator in the proliferation and migration of breast cancer cells by targeting E2F3. Thus, Brachyury/E2F3 axis might be used as a diagnosed biomarker. Our finding provides a novel mechanism of Brachyury biology and provides potential targets for the diagnosis of breast cancer.

## Data Availability

The Chip/PCR/Western Blot.etc data used to support the findings of this study are available from the corresponding author upon request.
